# Physical performance and chronic kidney disease development in elderly adults: results from a nationwide cohort study

**DOI:** 10.18632/aging.103741

**Published:** 2020-09-11

**Authors:** Young Su Joo, Jong Hyun Jhee, Hyung-Woo Kim, Seung Hyeok Han, Tae-Hyun Yoo, Shin-Wook Kang, Jung Tak Park

**Affiliations:** 1 Department of Internal Medicine, College of Medicine, Institute of Kidney Disease Research, Yonsei University, Seoul 03722, Republic of Korea; 2 Division of Nephrology, Department of Internal Medicine, Myongji Hospital, Goyang 10475, Gyeonggi-do, Republic of Korea; 3 Severance Biomedical Science Institute, Brain Korea 21 PLUS, Yonsei University, Seoul 03722, Republic of Korea; 4 Division of Nephrology, Department of Internal Medicine, Gangnam Severance Hospital, Yonsei University College of Medicine, Seoul 06273, Republic of Korea

**Keywords:** chronic kidney disease, elderly population, sarcopenia, frailty

## Abstract

Sarcopenia, which is characterized by muscle mass and physical performance, is closely associated with morbidities and mortality, especially among the elderly. However, the effect of physical performance on chronic kidney disease (CKD) development is not yet fully elucidated. A total of 30,871 adults aged 66 years with preserved renal function who underwent health screening examinations were evaluated. Physical performance was assessed using a 3-m timed up and go (TUG) test and the one-leg stand (OLS) test. The primary outcome was the development of CKD, defined as at least two consecutive measurements of estimated glomerular filtration rate < 60 mL/min/1.73 m^2^. The rates of mortality and incident CKD development were significantly elevated with increases in TUG test scores but not in OLS scores. In the Cox hazards model, the highest TUG test score tertile was associated with an increased risk for CKD development (hazard ratio, 1.23; 95% confidence interval, 1.10-1.38) compared with the lowest tertile. No significant relationship was observed between OLS score and incident CKD risk. Poor physical performance, assessed using the TUG test, was related to an increased risk of CKD development.

## INTRODUCTION

Chronic kidney disease (CKD) is one of the most common clinical problems among older people [1–3]. The CKD prevalence is reported to be as high as one-third to one-half of the elderly population [1, 4, 5]. With the improvement of life expectancy, the number of elderly patients with CKD is expected to increase much faster than at present. The increased prevalence of traditional risk factors, such as diabetes and hypertension, in the elderly is presumed to be the main cause of the pervasiveness of CKD in this population [6]. Nonetheless, the factors associated with CKD development in the elderly are not yet fully understood.

Sarcopenia is a syndrome characterized by involuntary generalized loss of skeletal muscle mass and strength [7]. Muscle mass and strength begin to decline as early as the third decade of life and progressively deteriorate with aging in a linear manner [8]. In a recent meta-analysis, the prevalence of sarcopenia was estimated to be about 10% of the population aged > 60 years worldwide when sarcopenia is defined by low muscle mass alone [9]. When taking muscle strength into consideration, up to 50% of muscle strength is reported to be lost by the eighth decade of life [10, 11]. Sarcopenia in the elderly is accompanied by loss of function, disability, and frailty [12–14]. However, previous studies have shown that the presence of sarcopenia also increases the risk of several chronic diseases such as insulin resistance and rheumatoid arthritis, as well as mortality risk [15–20].

Recently, in addition to muscle mass measurements, evaluation of muscle performance has been emphasized in detecting sarcopenia. The European Working Group on Sarcopenia in Older People (EWGSOP) recently recommended considering muscle performance in addition to muscle mass when defining sarcopenia [21]. Further, the Society of Sarcopenia, Cachexia, and Wasting Disorders published a definition that includes the measurement of walking speed [22]. Nonetheless, despite the prevalence of sarcopenia and CKD among the elderly, the relationship between physical performance and CKD development has not been widely evaluated.

Therefore, in this study, the association of physical performance, measured using the 3-m timed up and go (TUG) test and one-leg stand (OLS) test, with the development of incident CKD was investigated. This was done by evaluating a nationwide cohort of elderly health examinees.

## RESULTS

### Baseline characteristics

The baseline characteristics of the subjects according to tertiles of TUG and OLS test scores are shown in Table 1 and Supplementary Table 1, respectively. Among the 30,871 subjects, 15,070 (48.8%) were men. The mean estimated glomerular filtration rate (eGFR) was 84.9 ± 19.2 mL/min/1.73 m^2^. During the study period, more than 72.9% of subjects measured creatinine ≥4 times (Supplementary Figure 1). The median (interquartile range) TUG and OLS test scores were 9 (7-10) and 16 (10-20) seconds, respectively. The subjects in the higher TUG tertile groups tended to be women, have higher BMI, and frequently have a history of smoking. In addition, the proportions of subjects with chronic diseases including diabetes mellitus, hypertension, and cardiovascular disease (CVD) were elevated in tertile groups with higher TUG test scores. The proportions of subjects with dementia and malignancy were comparable among the groups. When the subjects were categorized according to OLS test scores, those in the higher OLS test score groups tended to be men, have lower BMI, and less likely have a history of CVD. Since baseline eGFR was lower in the lower TUG groups than the groups with higher TUG, the correlation between baseline eGFR and physical performance tests were examined using Pearson correlation analysis. The TUG test scores were positively associated with baseline eGFR (r=0.0321, *P*<.001) while OLS test scores did not show a significant correlation (Supplementary Figure 2).

**Table 1:** Baseline characteristics according to 3-m timed up and go test.

	**3-m timed up and go test tertile**			
	**Overall (N=30,871)**	**Tertile 1 (n=15,155)**	**Tertile 2 (n=10,067)**	**Tertile 3 (n=5649)**
**TUG test score, s**				
Mean	9.2 ± 4.1	6.9 ± 1.2	9.6 ± 0.5	14.9 ± 6.3
Median (IQR)	9 (7-10)	7 (6-8)	10 (9-10)	13 (12-15)
**OLS test score, s**				
Mean	16.9 ± 10.4	17.9 ± 10.6	16.6 ± 9.5	14.7 ± 11.1
Median (IQR)	16 (10-20)	18 (11-21)	15 (10-20)	13 (7-20)
**Demographic data**				
Male sex	15,070 (48.8)	7975 (52.6)	4698 (46.7)	2397 (42.5)
Body mass index, kg/m^2^	24.2 ± 3.0	24.1 ± 2.9	24.2 ± 3.0	24.4 ± 3.1
SBP, mmHg	130.1 ± 16.5	130.2 ± 16.6	129.8 ± 16.3	130.3 ± 16.7
DBP, mmHg	78.8 ± 10.2	78.8 ± 10.3	78.7 ± 10.1	79.1 ± 10.2
Smoking status				
Non-smoker	18,818 (61.0)	8893 (58.7)	6262 (62.2)	3663 (64.8)
Ex-smoker	.5946 (19.3)	3191 (21.1)	1833 (18.2)	922 (16.3)
Current smoker	.6107 (19.8)	3071 (20.3)	1972 (19.6)	1064 (18.9)
Drinker	13,533 (43.8)	6947 (45.8)	4319 (42.9)	2267 (40.2)
**Comorbidities**				
Diabetes mellitus	.6951 (22.5)	3240 (21.4)	2292 (22.8)	1419 (25.1)
Hypertension	15,602 (50.5)	7636 (50.4)	5022 (49.9)	2944 (52.1)
Arrhythmia	.2067 (6.7)	997 (6.6)	696 (6.9)	374 (6.6)
CVD	.5276 (17.1)	2394 (15.8)	1724 (17.1)	1158 (20.5)
Myocardial infarction	.602 (2.0)	306 (2.0)	192 (1.9)	104 (1.8)
Congestive heart failure	.1934 (6.3)	891 (5.9)	614 (6.1)	429 (7.6)
Peripheral arterial disease	.1086 (3.5)	473 (3.1)	376 (3.7)	237 (4.2)
Dementia	.460 (1.5)	213 (1.4)	149 (1.5)	98 (1.7)
Malignancy	.1937 (6.3)	953 (6.3)	629 (6.2)	358 (6.3)
**Laboratory parameters**				
eGFR, mL/min/1.73 m^2^	84.9 ± 19.2	84.4 ± 18.6	84.9 ± 19.3	86.3 ± 20.7
Glucose, mg/dL	101.3 ± 25.2	100.9 ± 23.9	101.5 ± 26.5	101.9 ± 26.4
Total cholesterol, mg/dL	198.3 ± 37.8	197.2 ± 37.0	199.0 ± 38.2	199.8 ± 38.9
HDL-C, mg/dL	53.7 ± 22.0	53.1 ± 17.6	53.8 ± 19.1	55.3 ± 33.9
Triglyceride, mg/dL	118 [85-168]	116 [84-166]	119 [85-168]	122 [87-173]

Note: All variables are expressed as mean and standard deviation, number and percentage, or median and 25^th^ and 75^th^ percentiles.

Cardiovascular disease (CVD) was defined as the presence of a history of myocardial infarction, coronary artery disease, congestive heart failure, peripheral artery disease, or cerebrovascular disease.

Abbreviations: TUG, 3-m timed up and go; OLS, one-leg stand; BMI, body mass index; SBP, systolic blood pressure; DBP, diastolic blood pressure; CVD, cardiovascular disease; eGFR, estimated glomerular filtration rate; HDL-C, high-density lipoprotein cholesterol.

### Outcomes

**Figure 1:**
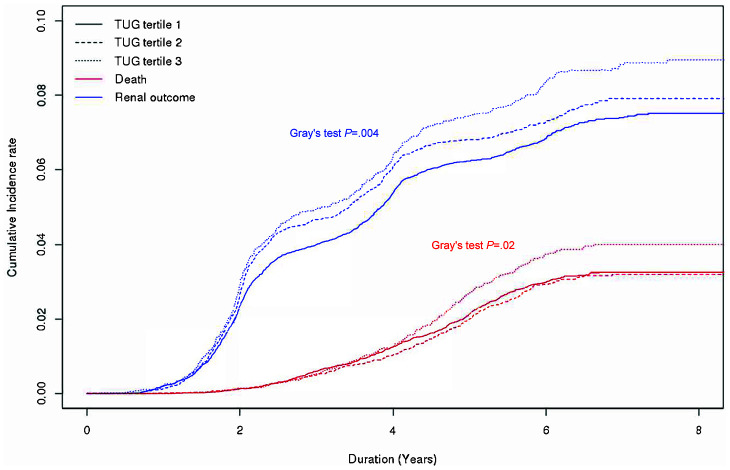
**Cumulative incidence curves for mortality and chronic kidney disease development according to 3-m timed up and go test tertile group.** Abbreviations: TUG, 3-m timed up and go test.

During a median of 6.0 (5.3-7.3) years and 181,627 person-years, there were 905 deaths of any cause and 2142 incident CKD events. The causes of mortality are shown in Supplementary Table 2. No significant association was found between specific mortality cause and physical activity. The incidence rate of CKD per 1000 person-years was 11.8 among the overall subjects. The corresponding rate of death was 5.0. The rates of CKD development and mortality were increased in the higher TUG tertile groups (*P*=.001 and *P*=.01, respectively) (Table 2). When the subjects were divided into tertiles of OLS test scores, the rate of incident CKD was comparable among the OLS tertile groups. Although the mortality rates significantly differed among the OLS tertile groups, no clear trend of change was observed (Supplementary Table 3). Similar results were observed when cumulative incidence function plots were constructed for mortality and CKD development according to TUG (Figure 1) and OLS tertile groups (Supplementary Figure 3).

**Table 2:** Outcome event rates according to 3-m timed up and go test tertile groups.

	**Overall**	**3-m timed up and go test**			
		**Tertile 1**	**Tertile 2**	**Tertile 3**	***P*^a^**
**Number of participants**	30,871	15,162	10,071	5667	
**Person-years**	181,627	89,254	59,148	33,225	
**Composite renal outcome**					
Events, n	2196	1019	722	455	
Incidence rate per 1000 person-years	12.1 (11.6-12.6)	11.4 (10.7-12.1)	12.2 (11.3-13.1)	13.7 (12.5-15.0)	.001
**Incident CKD**					
Events, n	2142	993	711	438	
Incidence rate per 1000 person-years	11.8 (11.3-12.3)	11.1 (10.5-11.8)	12.0 (11.2-12.9)	13.2 (12.0-14.5)	.002
**Incident ESRD**					
Events, n	65	28	16	21	
Incidence rate per 1000 person-years	0.36 (0.28-0.47)	0.31 (0.22-0.45)	0.27 (0.17-0.44)	0.63 (0.41-0.97)	. .03
**Death**					
Events, n	905	434	274	197	
Incidence rate per 1000 person-years	5.0 (4.7-5.3)	.4.9 (4.4-5.3)	.4.6 (4.1-5.2)	.5.9 (5.2-6.8)	. .01

Note: ^a^Log rank test.

Abbreviations: CKD, chronic kidney disease; ESRD, end-stage renal disease

### Associations between physical performance tests and CKD development

**Figure 2:**
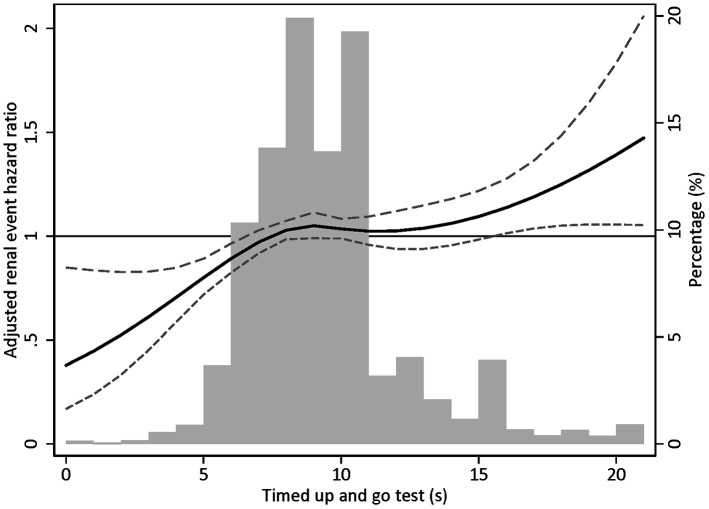
**Restricted cubic spline plot for incident chronic kidney disease according to 3-m timed up and go test.** Note: Adjusted for sex, estimated glomerular filtration rate, body mass index, systolic blood pressure, chronic obstructive pulmonary disease history, dementia history, diabetes mellitus history, cardiovascular disease, smoking habit, alcohol consumption, and high-density lipoprotein cholesterol.

In a cause-specific model adjusting for sex and baseline eGFR, the risk of incident CKD development was elevated by 23% in the highest TUG tertile group compared with the lowest TUG tertile group (hazard ratio [HR], 1.23; 95% confidence interval [95% CI], 1.10-1.38). This risk was attenuated but still statistically significant after adjusting for additional demographic and clinical variables (HR, 1.16; 95% CI, 1.03-1.30). Similar results were observed when the log-transformed TUG score was treated as a continuous variable (Table 3). In addition, in the subdistribution hazards model with death as a competing risk for incident CKD, the relationship between TUG test scores and CKD development risk were similar to the main association of the cause-specific hazards model (Table 4). However, when the subjects were grouped into OLS tertiles, there was no association between OLS test scores and incident CKD. Significant relationships between OLS test score and CKD development risk were also not found in the analysis using subdistribution hazards model (Supplementary Table 4). Spline analysis revealed a linear relationship between the TUG test score and incident CKD risk (Figure 2). However, no significant association was found between OLS test scores and renal outcome (Supplementary Figure 4). Sensitivity analyses excluding subjects with COPD, dementia, and CVD revealed similar findings, respectively (Supplementary Table 5). In addition, sensitivity analyses excluding those who have reached outcome within 2 years of physical performance test also showed results concordant to the findings of the main analysis (Supplementary Tables 6 and 7).

**Table 3:** Cause-specific hazard ratios according to 3-m timed up and go test for incident chronic kidney disease.

	**Model 1**		**Model 2**		**Model 3**	
	**HR (95% CI)**	***P***	**HR (95% CI)**	***P***	**HR (95% CI)**	***P***
**TUG score, per s**	1.32 (1.17-1.49)	<.001	1.28 (1.13-1.44)	<.001	1.24 (1.09-1.40)	<.001
**TUG score tertile**						
**1**	Ref		Ref		Ref	
**2**	1.11 (1.01-1.22)	...04	1.11 (1.01-1.22)	.03	1.11 (1.00-1.22)	.04
**3**	1.23 (1.10-1.38)	<.001	1.17 (1.04-1.31)	.008	1.16 (1.03-1.30)	.01

Note: TUG score and high-density lipoprotein cholesterol were log-transformed due to skewed distribution.

Model 1: adjusted for sex and estimated glomerular filtration rate.

Model 2: adjusted for model 2 plus body mass index, systolic blood pressure, chronic obstructive pulmonary disease history, dementia history, diabetes mellitus history, and cardiovascular disease history.

Model 3: adjusted for model 3 plus smoking habit, alcohol consumption, and high-density lipoprotein cholesterol.

Abbreviations: TUG; 3-m timed up and go; HR, hazard ratio; sHR, subdistribution hazard ratio; CI, confidence interval; Ref, reference.

**Table 4:** Subdistribution hazard ratios according to 3-m timed up and go test for incident chronic kidney disease with death as a competing event.

	**Model 1**		**Model 2**		**Model 3**	
	**sHR (95% CI)**	***P***	**sHR (95% CI)**	***P***	**sHR (95% CI)**	***P***
**TUG score, per s**	1.30 (1.13-1.48)	<.001	1.26 (1.10-1.44)	<.001	1.23 (1.07-1.41)	.004
**TUG score tertile**						
**1**	Ref		Ref		Ref	
**2**	1.11 (1.00-1.23)	.06	1.10 (0.99-1.22)	.07	1.10 (0.99-1.22)	.07
**3**	1.23 (1.08-1.39)	.001	1.17 (1.04-1.33)	.01	1.15 (1.01-1.29)	.03

Note: TUG score and high-density lipoprotein cholesterol were log-transformed due to skewed distribution.

^a^In Fine-Gray model, mortality was considered as a competing risk.

Model 1: adjusted for sex and estimated glomerular filtration rate.

Model 2: adjusted for model 2 plus body mass index, systolic blood pressure, chronic obstructive pulmonary disease history, dementia history, diabetes mellitus history, and cardiovascular disease history.

Model 3: adjusted for model 3 plus smoking habit, alcohol consumption, and high-density lipoprotein cholesterol.

Abbreviations: TUG; 3-m timed up and go; HR, hazard ratio; sHR, subdistribution hazard ratio; CI, confidence interval; Ref, reference.

### Subgroup analysis

To evaluate the modification effects of subgroups on the relationship between physical performance and incident CKD risk, subgroup analyses were performed in subgroups stratified by sex, BMI, diabetes, hypertension, and CVD (Supplementary Figures 5 and 6). The association between physical performance and incident CKD risk was similar to that in the main analysis across these subgroups.

## DISCUSSION

In this study, the association between physical performance and risk of incident CKD development was evaluated in an elderly population with normal renal function. This was performed by analyzing data from a nationwide health screening examination cohort. The TUG and OLS tests, which are generally utilized for sarcopenia evaluation, were used as surrogate measurements of physical performance. The incidences of CKD and death gradually increased with higher TUG test scores. In addition, higher TUG test scores were significantly associated with the development of CKD even after adjustments for multiple confounding factors. Moreover, the significance of this relationship was maintained in sensitivity analyses using subdistribution hazards models. However, a significant relationship between OLS test score and CKD development risk was not found. These results suggest that poor physical function, evaluated using the TUG test, could be a risk factor for CKD development in the elderly.

Previous investigations have reported significant associations between physical function and the risk of chronic disease development. Low gait speed was reported to be associated with increased risk of CVD in the elderly population as well as in dialysis-dependent CKD patients [23–26]. In addition, evaluation of the Women’s Health and Aging Study showed that physical function predicts catastrophic disability in activities of daily living [27]. Moreover, slow gait speed was reported to be a significant risk factor for mortality among elderly Italians [28]. In addition to physical performance status being a risk factor for chronic diseases, physical performance also predicts outcome in several patient groups. A recent meta-analysis involving 4187 cancer patients showed that physical performance is closely related to survival and suggested that physical performance tests should be used as a prognostic tool in patients with cancer [29]. In patients with Alzheimer’s disease, physical performance was found to be significantly related to future cognitive function [30]. In coronary artery disease patients, physical performance was reported to predict the occurrence of future cardiovascular events [31]. Adding to the relationships of physical function with poor outcome, the results of this study showed that poor physical function may also increase the probability of kidney function loss. The findings suggest that TUG tests could be used as a prognostic factor for kidney function decline in the elderly.

Although a clear association between TUG test score and incident CKD was observed, no significant relationship was found between OLS test score and CKD development. Several previous reports have also provided inconsistent findings on the association between OLS tests and adverse events. In a Japanese population of 80-year-old subjects, OLS test scores were not associated with overall mortality or specific causes of mortality such as CVD, pneumonia, and malignancy [32]. Investigations examining the relation between OLS test score and falling events also provided variable results. Although several studies found a clear difference in OLS test scores between fallers and non-fallers, others failed to report such a difference. One of the reasons for this weak clinical relevance of the OLS test score could be related to its poor correlation with muscle mass. A recent investigation reported that OLS test scores did not show an independent correlation with leg muscle mass in older women [33]. In addition, the fact that the OLS test cannot be conducted in subjects with cognitive impairment or in those using a walking aid further limits its suitability as a performance measurement in elderly people. Concordantly, when predicting fall events among community-dwelling older people, the predictive value of TUG test scores exceeded that of OLS test scores in a recent investigation [34].

Although the eGFR was within the normal range in all TUG score groups, the TUG test scores were positively associated with baseline eGFR. One of the reasons for this relationship between TUG test score and eGFR could be the possibility of overestimation. TUG test scores are known to be related with muscle mass in the elderly [35]. Since serum creatinine levels are affected by muscle mass, eGFR is often overestimated in patients with lower muscle mass [36]. A similar finding was also observed in a recent cross-sectional study among older people. In that study, the TUG was lower in subjects with eGFR 60-89 ml/min/1.73m^2^ compared to subjects with eGFR > 90ml/min/1.73m^2^ which is a finding comparable to the results of the current investigation [37]. In order to allow for this relationship between TUG score and eGFR at baseline, the risk for incident CKD was adjusted for baseline eGFR in the multivariable hazards models.

Several mechanisms may be accountable for explaining the association between TUG test score and CKD development. Gait speed, assessed using a 400-m walk test, was reported to be inversely associated with circulating inflammatory markers such as fibrinogen, C-reactive protein, and interleukin-6 [38, 39]. As chronic systemic inflammation is a well-known risk factor for CKD development, inflammatory cytokines could be a factor linking physical function and CKD. This possibility could not be verified in this study because the health screening examinations conducted by the National Health Insurance Service (NHIS) do not include tests for inflammatory markers such as C-reactive protein. Further investigations encompassing data on systemic inflammation would be needed. In addition, metabolic derangements could have mediated kidney function deterioration. Several reports have shown a clear connection between insulin resistance and loss of muscle mass in the elderly [40–42]. Accordingly, diabetes was more prevalent among those with higher TUG test scores in this study. However, as the association between TUG test score and CKD remained robust even after adjusting for diabetes, factors other than metabolism may have played roles.

This study has several strengths. First, the current study used a nationwide representative sample, which encompasses health examination results of >30,000 individuals, enhancing the possibility that the results can be generalized to the general population. Second, the relatively long follow-up duration of 8 years is advantageous in detecting slowly progressing chronic diseases such as CKD. Finally, the cohort consisted of a specific age group (subjects aged 66 years). This minimized the confounding effect of age, which is the most important factor affecting physical performance and kidney function. The findings of this study should also be interpreted in light of the study limitations. First, limitations due to the sampled cohort study design were inevitable. However, the NHIS-National Sample Cohort was constructed using a population-based sampling strategy, which has been designed that the sample cohort truly represents the whole data set, reducing the possibility of any selection bias [43]. Second, limitations related with medical insurance claims data should be addressed. The cost of renal replacement therapy is covered by the National Health Insurance, a mandatory insurance for all Koreans. Therefore, the chances of the insurance claims data to misidentify ESRD outcomes would be low. The fact that the ESRD incidence rate (0.36 per 1000 person-year) in this study was somewhat higher than the annual ESRD prevalence reported by the Korean Society of Nephrology (KSN) ESRD registry data (0.25 per 1000 person), where registration is not mandatory, supports this notion [44]. However, comorbidities other than ESRD that do not require active health care services may not have been precisely identified by the claims data. In addition, the possibility of documentation error would also be inevitable in dealing with large sized administrative data. Moreover, influential information such as family history and comorbidity duration were not available due to the nature of the data source. Third, a confirmative causal link between TUG score and CKD development cannot be concluded owing to the retrospective design of the investigation. Further prospective evaluations of the effect of physical performance improvement on kidney function would be needed. Fourth, although biennial health examinations are the minimal recommendation of the NHIS for all beneficiaries, a potential bias exists. CKD development can be detected only when the subjects undergo periodic health examinations. Some of the subjects could have skipped their biannual health examinations. Finally, physical performance tests were conducted only once at baseline. Therefore, dynamic physical performance during the follow-up period was not taken into account.

In this study, the risk of CKD development was found to be related to poor physical function in relatively healthy elderly adults. Poor TUG test scores were associated with both CKD development and mortality, whereas OLS test scores did not show significant relationships. These results suggest that poor physical performance, especially assessed through TUG test, could be a risk factor for kidney function decline in the elderly. However, further investigations clarifying the causal relationship between physical performance and incident CKD are warranted.

## MATERIALS AND METHODS

### Data source and study subjects

**Figure 3:**
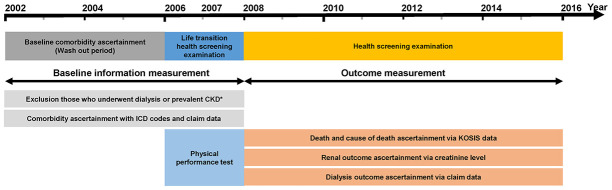
**Design of the study.** Note: Data from the National Health Insurance Service-National Sample Cohort Database were used. Abbreviations: ICD-10, 10^th^ revision of the International Statistical Classification of Diseases and Related Health Problems.

Detailed information regarding data source are described in the Supplementary Methods. The present study was conducted in accordance with the Declaration of Helsinki and approved by the institutional review board of Yonsei University Health System Trial Center (approval no. 4-2018-0697). The informed consent requirement was waived owing to the retrospective nature of the analysis. Data usage was also approved by the national health information data request review committee of NHIS. According to the Act on the Protection of Personal Information Maintained by Public Institutions, the NHIS provides data on health examination results and detailed medical treatments after de-identification of individual-level data. Data were retrieved from the National Health Insurance Service-National Sample Cohort Database (NHIS-NSC DB), which is a retrospective population-based sample cohort constructed on a 2.2% representative sample of the Korean population. The detailed cohort profiles with respect to the development of the NHIS-NSC DB have been previously published [43]. Data acquired during the life transition health screening examination at age 66 years were considered as baseline information. The health screening examination results obtained thereafter were used as follow-up data (Figure 3).

Elderly adults who underwent the life transition health screening examination between 2007 and 2008 were initially screened for enrollment (N=42,132). Subjects who met the following criteria were excluded: (1) eGFR <60 mL/min/1.73 m^2^, presence of proteinuria in dipstick urine examination at baseline visit, or history of any renal replacement therapy including renal transplantation and dialysis (2) missing data on TUG test, OLS test, follow-up health screening examinations, or lifestyle questionnaires that include information on smoking and alcohol use. A total of 30,871 subjects were included in the final analysis (Supplementary Figure 7).

### Data collection and measurements

Baseline demographic data, including demographic and physical function tests and anthropometric measurements, were collected during the life transition health screening examination. Blood samples were obtained after ≥8-h fasting. Serum creatinine level was measured using an isotope dilution mass spectrometry-calibrated method. eGFR was calculated using the creatinine-based CKD Epidemiology Collaboration equation [45]. Urine samples were collected in the morning after the first voiding. Urine tests were performed on fresh urine samples with urine reagent strips, using regularly calibrated semi-automatic urine analyzers. Urine protein amounts were determined as absent, trace, 1+, 2+, or 3+, which approximately correlate with urine protein levels of <10, 10-20, >30, >100, and >500 mg/dL, respectively. Proteinuria was considered present when the urinalysis result was higher than trace level. Comorbidities during the year before the baseline examination were defined based on *International Classification of Diseases, Tenth Revision* (ICD-10) codes and claim records. Hypertension was defined as follows: (1) systolic blood pressure >140 mmHg or diastolic blood pressure >90 mmHg at baseline examination, (2) one or more ICD codes (I10-13 or I14) with antihypertensive claim data before baseline examination, and (3) two or more ICD codes (I10-13 or I14) before baseline examination. Diabetes mellitus was defined as the presence of one inpatient E11-E14 codes (ICD-10) or >2 outpatient E11-E14 codes in claim data or a diabetes drug code with E11-E14 codes. The detailed operational definitions of comorbidities are provided in Supplementary Table 8.

### Exposure and outcome ascertainment

TUG and OLS tests were performed by trained examining physicians. For the TUG test, the time taken to get up from a standard armchair, walk a 3-m distance, turn around, and return to sitting in the chair was recorded [46]. For the OLS test, participants had to stand on one leg for as long as possible with the contralateral leg not bearing weight. The time until balance was lost or until the non-weight-bearing leg touched the floor was recorded [47].

The primary outcome was incident CKD and the secondary outcome was death of any cause. Incident CKD was defined as at least two consecutive measurements of eGFR <60 mL/min/1.73 m^2^ during follow-up health examinations. Deaths and cause of death were ascertained from records that were linked with the Korean Statistical Information Service using unique personal identification numbers [48]. Subjects were censored at the date of the last health examination, the development of CKD, death, or the end of the study period (December 31, 2015), whichever occurred first.

### Statistical analysis

Detailed information regarding statistical analysis are described in the Supplementary Method. All analyses were performed using Stata version 15.1 (Stata Corp, College Station, TX, USA), R software 3.3.3 (http://www.R-project.org), and SAS version 9.4 (SAS Institute Inc., Cary, NC, USA). The TUG and OLS test scores were analyzed as continuous variables, and tertiles of the test scores were analyzed as categorical variables. Cumulative incidence function was used to estimate the cumulative outcome curves, and the homogeneity of the each survival curve was evaluated using Gray’s test [49]. To evaluate the association between the physical performance tests and incident CKD, multistep multivariable proportional cause-specific hazards models were constructed. Death before reaching the primary outcome was considered as a competing outcome and censored at the time of death [50, 51]. Covariates hypothesized to contribute to renal function deterioration were included in the adjusted models. In model 1, sex and baseline eGFR were adjusted. Model 2 additionally adjusted for BMI, systolic blood pressure, chronic obstructive pulmonary disease (COPD) history, dementia history, diabetes, and CVD history. Finally, further adjustments were made for smoking history, alcohol consumption, and high-density lipoprotein cholesterol in model 3. Subgroup analyses were performed according to sex, BMI, diabetes, hypertension, and CVD. For sensitivity analysis, the cause-specific models were analyzed after excluding subjects with comorbid diseases that can affect physical performance tests, including CVD, COPD, and dementia. In addition, the association of physical function test results and development of CKD in Fine-Gray models was evaluated [52]. The covariates that were adjusted in the cause-specific hazards models were used in the sensitivity analysis. All tests were two sided. All *P*-values <.05 were considered statistically significant.

## Supplementary Material

Supplementary Methods

Supplementary Figures

Supplementary Tables
