# Supplementary material for: Physical performance and chronic kidney disease development in elderly adults: results from a nationwide cohort study
Source: Aging (Albany NY). 2020 Sep 11;12(17):17393–417. doi: 10.18632/aging.103741 (PMC7521486; doi:10.18632/aging.103741)
Supplement: Supplementary Methods [file aging-12-103741-s002..pdf]

SUPPLEMENTARY METHODS

Data source

Data were retrieved from the National Health Insurance Service-National Sample Cohort Database (NHIS-NSC DB), which is a retrospective population-based sample cohort constructed on a 2.2% representative sample of Koreans. Korea has a single mandatory health insurance system, the National Health Insurance Service (NHIS), which maintains records of all covered medical visits, procedures, prescriptions, and health screening examinations. The detailed cohort profiles with respect to the development of the NHIS-NSC DB have been previously published [1].

The NHIS-NSC DB consists of the following data sets: (1) sociodemographic information and year and cause of death of the insurance beneficiary, which is reported from the Korean Statistical Information Service (KOSIS); (2) information on diagnosis based on *International Classification of Diseases, Tenth Revision* (ICD-10) codes, hospital admission, and treatment details including prescription of drugs and procedures; and (3) health screening examination data. The NHIS provides biannual health screening examinations that include laboratory tests, questionnaires for assessing cardiovascular risk factors, and anthropometric measurements (e.g., body weight, blood pressure, and waist circumference) to all of its beneficiaries. In addition to the routine biannual health screening examination, examinees who turn 66 years of age undergo a "life transition health screening examination", which consists of the TUG and OLS tests for the evaluation of muscle function. Data acquired during the life transition health screening examination at age 66 years were considered as baseline information. The health screening examination results obtained thereafter were used as follow-up data.

Statistical analysis

All analyses were performed using Stata version 15.1 (Stata Corp, College Station, TX, USA), R software 3.3.3 (http://www.R-project.org), and SAS version 9.4 (SAS Institute Inc., Cary, NC, USA). Continuous variables are expressed as either mean ± standard deviation or median (interquartile range), and categorical variables are presented as number (percentage). To test whether the variables are normally distributed, the Kolmogorov-Smirnov test was used. Analysis of variance and χ^2 tests were used to compare the difference between categorized groups for continuous and categorical variables, respectively. The Kruskal-Wallis test was used to determine the difference between groups when the variable showed a skewed distribution. The TUG and OLS test scores were analyzed as continuous variables, and tertiles of the test scores were analyzed as categorical variables. Cumulative incidence function was used to estimate the cumulative outcome curves, and the homogeneity of the each survival curve was evaluated using Gray's test [2]. To evaluate the association between the physical performance tests and incident CKD, multistep multivariable proportional cause-specific hazards models were constructed. Death before reaching the primary outcome was considered as a competing outcome and censored at the time of death [3, 4]. The result of cause-specific hazards models were presented as HRs and 95% CIs. Covariates hypothesized to contribute to renal function deterioration were included in the adjusted models. In model 1, sex and baseline eGFR were adjusted. Model 2 additionally adjusted for BMI, systolic blood pressure, chronic obstructive pulmonary disease (COPD) history, dementia history, diabetes, and CVD history. Finally, further adjustments were made for smoking history, alcohol consumption, and high-density lipoprotein cholesterol in model 3. Subgroup analyses were performed according to sex, BMI, diabetes, hypertension, and CVD. For sensitivity analysis, the cause-specific models were analyzed after excluding subjects with comorbid diseases that can affect physical performance tests, including CVD, COPD, and dementia. In addition, the association of physical function test results and development of CKD in Fine-Gray models was evaluated [5]. The covariates that were adjusted in the cause-specific hazards models were used in the sensitivity analysis. All tests were two sided. All *P*-values <.05 were considered statistically significant.

REFERENCES

1. Lee J, Lee JS, Park SH, Shin SA, Kim K. Cohort Profile: The National Health Insurance Service-National Sample Cohort (NHIS-NSC), South Korea. Int J Epidemiol. 2017; 46:e15. https://doi.org/10.1093/ije/dyv319 PMID:26822938

2. Gray RJ. A Class of K-Sample Tests for Comparing the Cumulative Incidence of a Competing Risk. Ann Stat. 1988; 16:1141–1154. https://doi.org/10.1214/aos/1176350951

3. Noordzij M, Leffondré K, van Stralen KJ, Zoccali C, Dekker FW, Jager KJ. When do we need competing risks methods for survival analysis in nephrology? Nephrol Dial Transplant. 2013; 28:2670–7. https://doi.org/10.1093/ndt/gft355 PMID:23975843

4. Andersen PK, Geskus RB, de Witte T, Putter H. Competing risks in epidemiology: possibilities and pitfalls. Int J Epidemiol. 2012; 41:861–70. https://doi.org/10.1093/ije/dyr213 PMID:22253319

5. Fine JP, Gray RJ. A Proportional Hazards Model for the Subdistribution of a Competing Risk. *J Am Stat Assoc.* 1999;94:496–509. https://doi.org/10.1080/01621459.1999.10474144
