# Supplementary material for: Physical performance and chronic kidney disease development in elderly adults: results from a nationwide cohort study
Source: Aging (Albany NY). 2020 Sep 11;12(17):17393–417. doi: 10.18632/aging.103741 (PMC7521486; doi:10.18632/aging.103741)
Supplement: Supplementary Figures [file aging-12-103741-s003..pdf]

SUPPLEMENTARY FIGURES

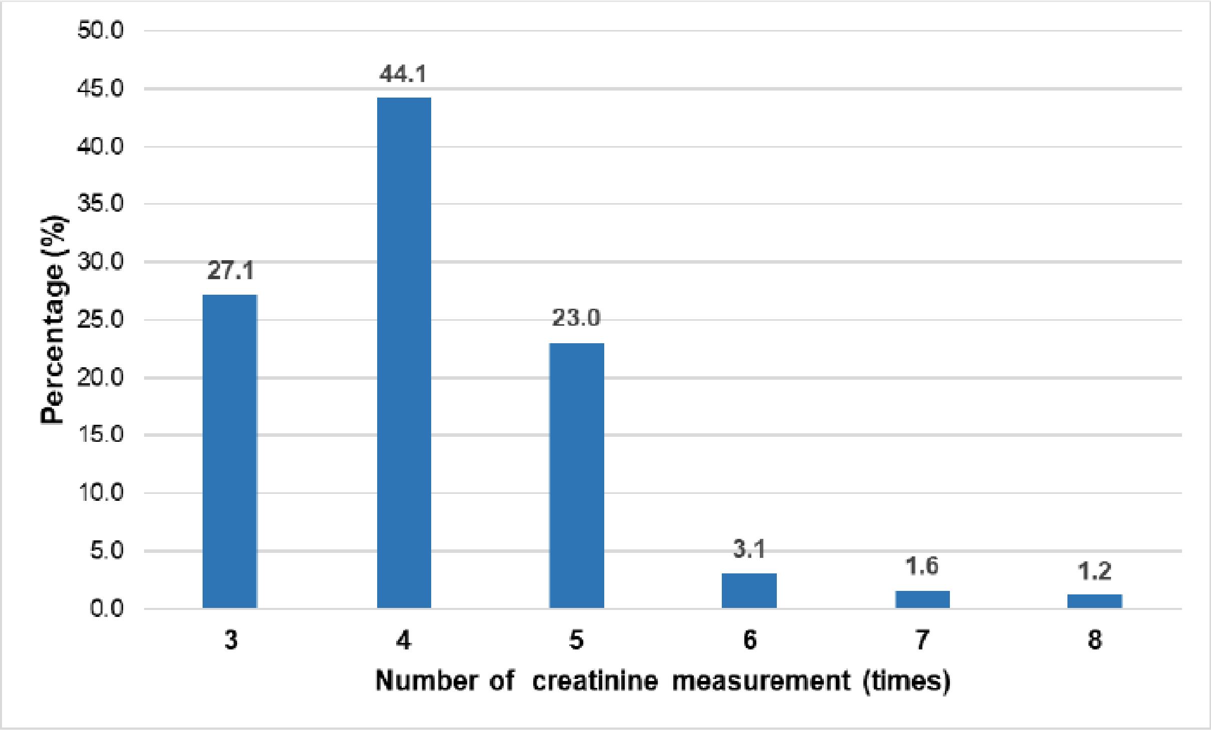

Supplementary Figure 1. The frequency of creatinine measurement during the study period.

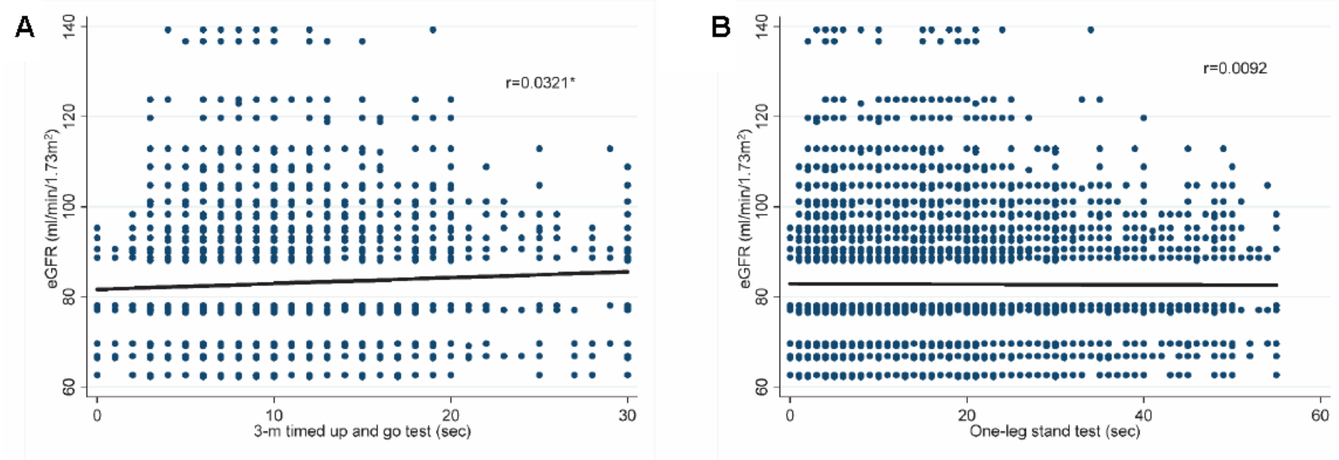

Supplemental Figure 2. Correlation between baseline estimated glomerular filtration rate and physical performance tests for (**A**) 3-m timed up and go test and (**B**) One-leg stand test. *Note:* Pearson correlation coefficient (*P*-value) of 3-timed up and go test and one-leg stand test were 0.0321 (<.001) and 0.0092 (.71), respectively. **P*<.05.

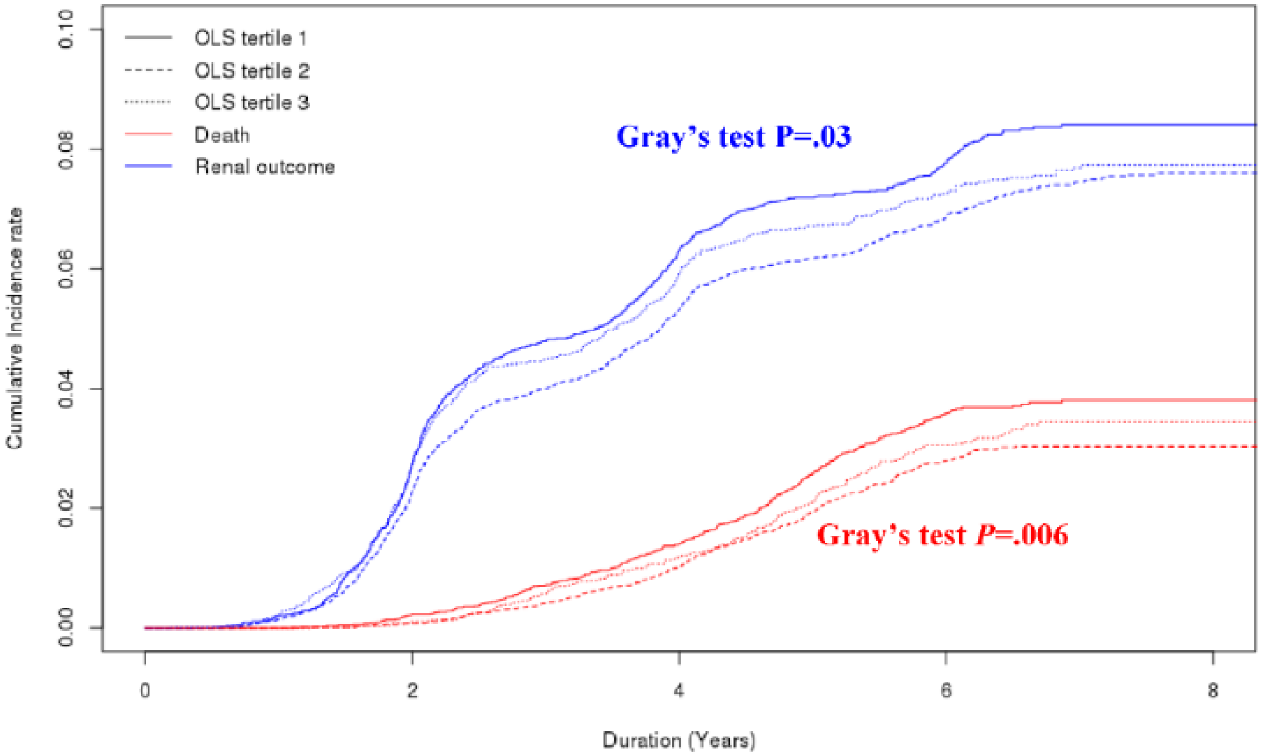

Supplementary Figure 3. Cumulative incidence curves for mortality and chronic kidney disease development according to one-leg stand test tertile group. *Abbreviations:* OLS, one-leg stand test.

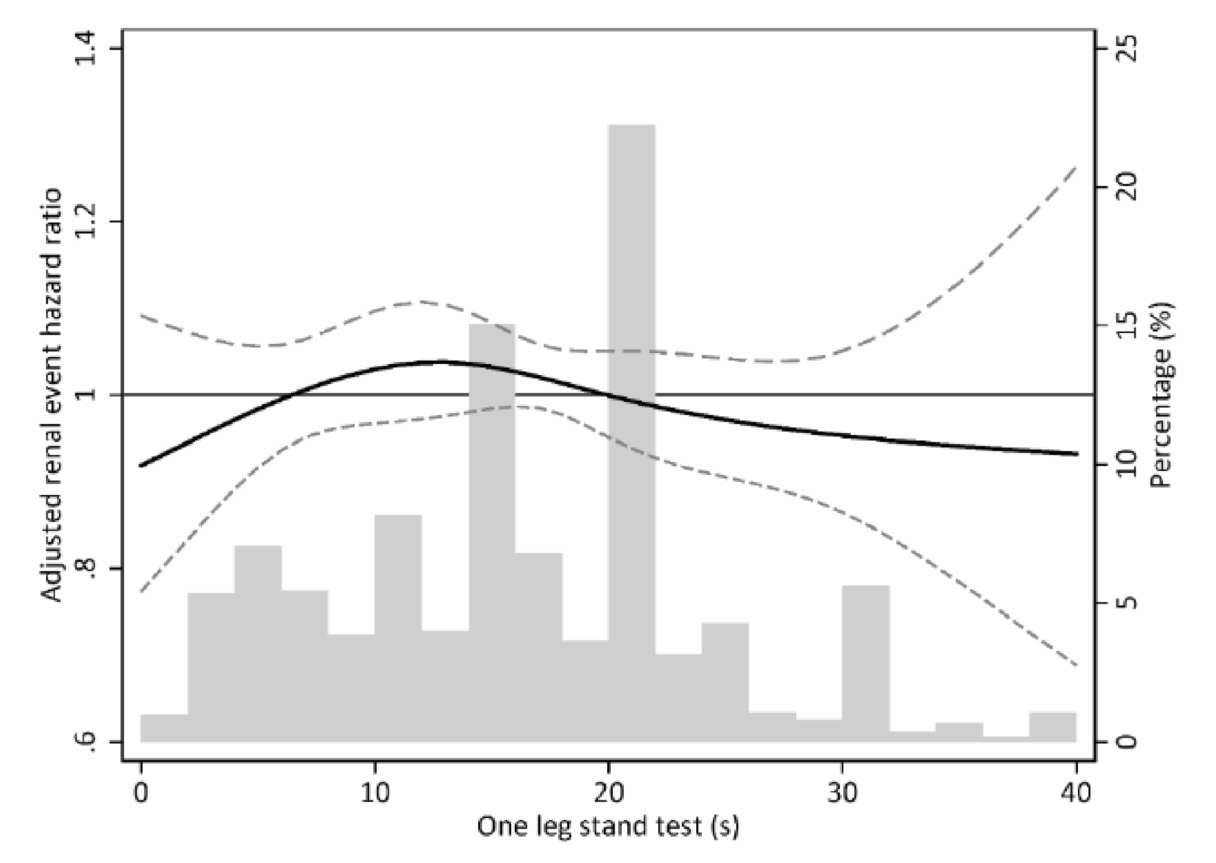

Supplementary Figure 4. Restricted cubic spline plot for incident chronic kidney disease according to one-leg stand test. *Note:* Adjusted for sex, estimated glomerular filtration rate, body mass index, systolic blood pressure, chronic obstructive pulmonary disease history, dementia history, diabetes mellitus history, cardiovascular disease, smoking habit, alcohol consumption, and high-density lipoprotein cholesterol.

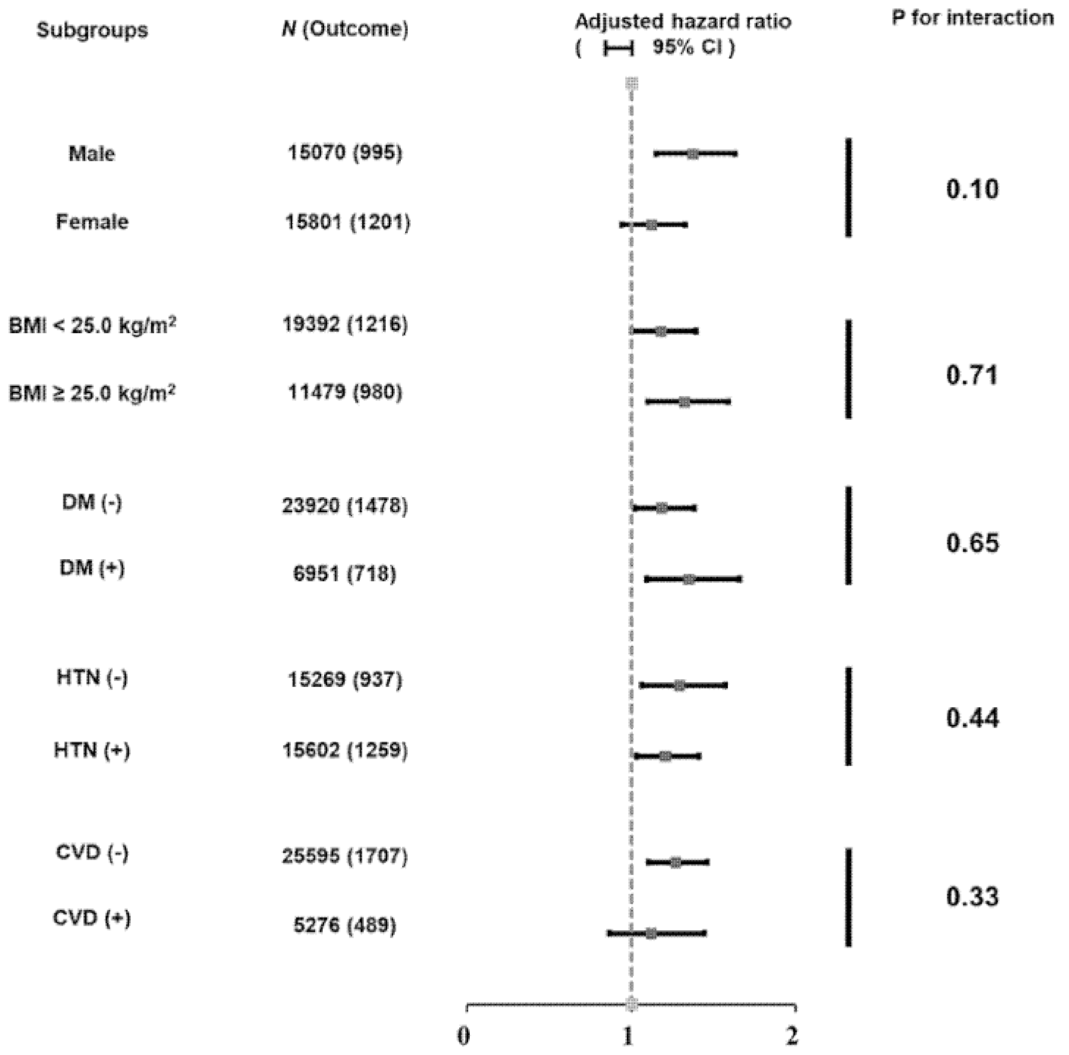

Supplementary Figure 5. Subgroup analysis of the 3-m timed up and go test for incident chronic kidney disease. *Note:* The 3-m timed up and go test score was log-transformed due to skewed distribution. Adjusted for sex, estimated glomerular filtration rate, body mass index, systolic blood pressure, chronic obstructive pulmonary disease history, dementia history, diabetes mellitus history, cardiovascular disease, smoking habit, alcohol consumption, and high-density lipoprotein cholesterol. *Abbreviations:* CI, confidence interval; BMI, body mass index; DM, diabetes mellitus; HTN, hypertension, CVD; cardiovascular disease.

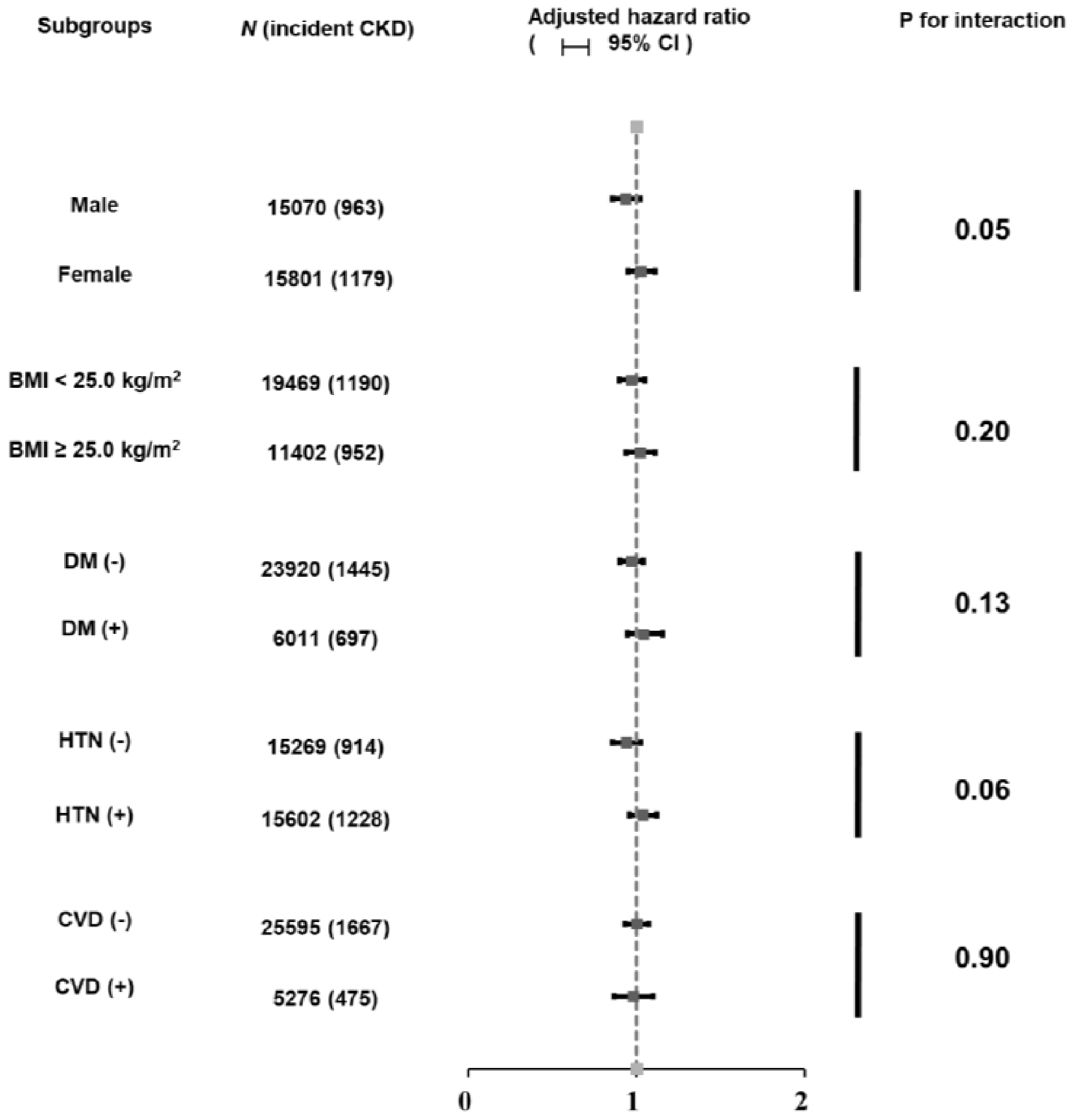

Supplementary Figure 6. Subgroup analysis of one-leg stand test for incident chronic kidney disease. *Note:* one-leg stand test score was log-transformed due to skewed distribution. Adjusted for sex, estimated glomerular filtration rate, body mass index, systolic blood pressure, chronic obstructive pulmonary disease history, dementia history, diabetes mellitus history, and cardiovascular disease history, smoking habit, alcohol consumption, and high density lipoprotein cholesterol. *Abbreviations:* CKD, chronic renal disease; CI, Confidence interval.

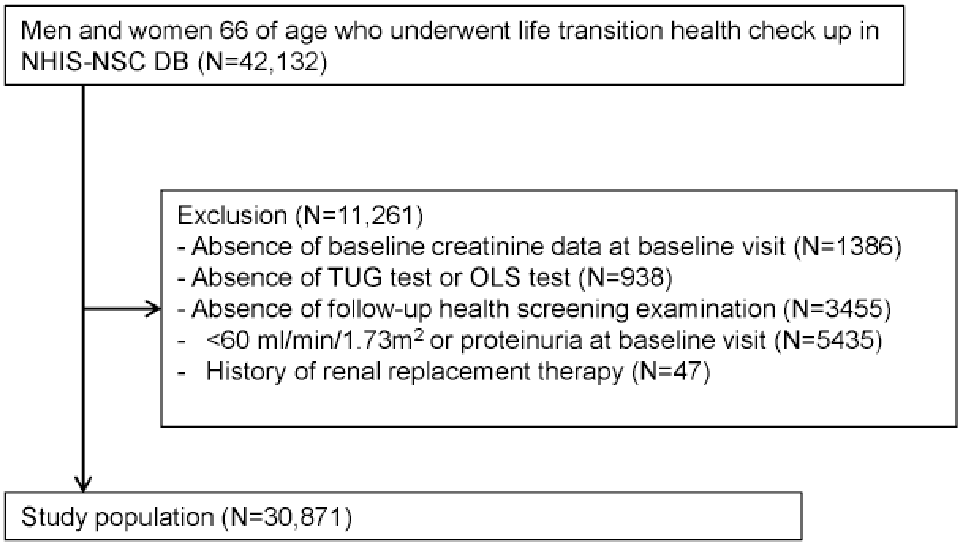

Supplementary Figure 7. Flowchart of subject selection. *Abbreviations:* NHIS-NSC DB, National Health Insurance Service-National Sample Cohort Database; TUG, 3-m timed up and go; OLS, one-leg stand.
