# Supplementary material for: Physical performance and chronic kidney disease development in elderly adults: results from a nationwide cohort study
Source: Aging (Albany NY). 2020 Sep 11;12(17):17393–417. doi: 10.18632/aging.103741 (PMC7521486; doi:10.18632/aging.103741)
Supplement: Supplementary Tables [file aging-12-103741-s001..pdf]

SUPPLEMENTARY TABLES

Supplementary Table 1. Baseline characteristics according to one-leg stand test.

		One-leg stand test tertile		
	**Overall (n=30,871)**	**Tertile 1 (n=10,476)**	**Tertile 2 (n=13,478)**	**Tertile 3 (n=6,917)**
**OLS test score, s**				
Mean	16.9 ± 10.4	7.1 ± 3.4	17.6 ± 2.3	30.4 ± 11.4
Median (IQR)	16 (10-20)	7 (4-10)	18 (15-20)	28 (23-30)
**Demographic data**				
Male sex	15,070 (48.8)	4,170 (39.8)	6,894 (51.2)	4,006 (57.9)
Body mass index, kg/m2	24.2 ± 3.0	24.5 ± 3.1	24.1 ± 2.9	23.9 ± 2.8
SBP, mmHg	130.1 ± 16.5	130.8 ± 16.8	129.7 ± 16.3	129.9 ± 16.2
DBP, mmHg	78.8 ± 10.2	79.1 ± 10.3	78.7 ± 10.1	78.8 ± 10.2
Smoking status				
Non-smoker	18,818 (61.0)	7,046 (67.3)	7,974 (62.2)	3,798 (54.9)
Ex-smoker	5,946 (19.3)	1,654 (15.8)	2,718 (20.2)	1,574 (22.8)
Current smoker	6,107 (19.8)	1,776 (17.0)	2,786 (20.7)	1,545 (22.3)
Drinker	13,533 (43.8)	4,078 (38.9)	6,120 (45.4)	3,335 (48.2)
**Comorbidities**				
Diabetes mellitus	6,951 (22.5)	2,637 (25.2)	2,821 (20.9)	1,493 (21.6)
Hypertension	15,602 (50.5)	5,545 (52.9)	6,653 (49.4)	3,404 (49.2)
Arrhythmia	2,067 (6.7)	725 (6.9)	883 (6.6)	459 (6.6)
CVD	5,276 (17.1)	2,046 (19.5)	2,165 (16.1)	1,065 (15.4)
Myocardial infarction	602 (2.0)	220 (2.1)	263 (2.0)	119 (1.7)
Congestive heart failure	1,934 (6.3)	770 (7.4)	776 (5.8)	388 (5.6)
Peripheral arterial disease	1,086 (3.5)	356 (3.4)	475 (3.7)	255 (3.7)
Dementia	460 (1.5)	174 (1.7)	190 (1.4)	96 (1.4)
Malignancy	1,937 (6.3)	653 (6.2)	850 (6.3)	434 (6.3)
**Laboratory parameters**				
eGFR, mL/min/1.73m^2	84.9 ± 19.2	85.5 ± 19.9	84.1 ± 18.2	85.5 ± 20.0
Glucose, mg/dL	101.3 ± 25.2	101.9 ± 26.2	101.2 ± 25.0	100.6 ± 24.2
Total cholesterol, mg/dL	198.3 ± 37.8	200.1 ± 38.3	197.7 ± 37.8	199.8 ± 38.9
HDL-C, mg/dL	52 [44-61]	52[44-61]	52 [44-60]	51 [43-61]
Triglyceride, mg/dL	118 [85-168]	121 [87-172]	117 [83-165]	116 [84-166]

Note: All variables are expressed as mean and standard deviation, number and percentage, or median and 25th and 75th percentiles

Cardiovascular disease (CVD) was defined as the presence of a history of myocardial infarction, coronary artery disease, congestive heart failure, peripheral artery disease, or cerebrovascular disease.

Abbreviations: OLS, one-leg stand; BMI, body mass index; SBP, systolic blood pressure; DBP, diastolic blood pressure; CVD, cardiovascular disease; eGFR, estimated glomerular filtration rate; HDL-C, high-density lipoprotein cholesterol

Supplementary Table 2. Cause of death according to physical performance groups.

Cause of death, n (%)	Overall	Tertile 1	Tertile 2	Tertile 3	*P*
**Timed up and go test**					.8
Malignancy	446 (49.3)	223 (51.5)	133 (48.5)	90 (45.7)	
Cardiovascular disease	153 (16.9)	77 (17.8)	45 (16.4)	31 (15.7)	
Unintentional injury	137 (15.2)	59 (13.6)	44 (16.1)	34 (17.3)	
Respiratory disease	52 (5.8)	22 (5.1)	16 (5.8)	14 (7.1)	
Gastroenteric disease	26 (2.9)	11 (2.5)	9 (3.3)	6 (3.0)	
Endocrinologic disease	21 (2.3)	9 (2.1)	6 (2.2)	6 (3.0)	
Infective disease	13 (1.4)	5 (1.2)	7 (2.6)	1 (0.5)	
Kidney disease	4 (0.4)	3 (0.7)	0 (0.0)	1 (0.5)	
Etc	52 (5.8)	24 (5.5)	14 (5.1)	14 (7.1)	
**One leg stand test**					.2
Malignancy	446 (49.3)	146 (42.2)	196 (55.2)	104 (51.2)	
Cardiovascular disease	153 (16.9)	62 (17.9)	58 (16.3)	33 (16.3)	
Unintentional injury	137 (15.2)	62 (17.9)	44 (12.4)	31 (15.3)	
Respiratory disease	52 (5.8)	21 (6.1)	17 (4.8)	14 (6.9)	
Gastroenteric disease	26 (2.9)	12 (3.5)	9 (2.5)	5 (2.5)	
Endocrinologic disease	21 (2.3)	9 (2.6)	8 (2.3)	4 (2.0)	
Infective disease	13 (1.4)	5 (1.4)	7 (2.0)	1 (0.5)	
Kidney disease	4 (0.4)	1 (0.3)	1 (0.3)	2 (1.0)	
Etc	52 (5.8)	28 (8.1)	15 (4.2)	9 (4.4)	

Supplementary Table 3. Outcome event rates according to one-leg stand test tertile groups.

	Overall	One leg stand test			
		Tertile 1	Tertile 2	Tertile 3	*P*[a]
**N**	30,871	10,476	13,478	6,917	
**Person-years**	181,647	60,299	80,501	40,847	
**Incident CKD**					
Events, n	2,142	767	896	479	
Incidence rate per 1000 person-years	11.8 (11.3-12.3)	12.7 (11.9-13.7)	11.1 (10.4-11.9)	11.7 (10.7-12.8)	.05
**Death**					
Events, n	905	346	355	204	
Incidence rate per 1000 person-years	5.0 (4.7-5.3)	5.7 (5.2-6.4)	4.4 (4.0-4.9)	5.0 (4.4-5.7)	.002

Note: [a]Log rank test

Abbreviations: CKD, chronic kidney disease

Supplementary Table 4. Hazard ratios for incident chronic kidney disease according to one-leg stand test.

	Model 1		Model 2		Model 3	
	**HR (95% CI)**	***P***	**HR (95% CI)**	***P***	**HR (95% CI)**	***P***
**Cause-specific model**						
**OLS score, per s**	0.94 (0.88-0.99)	.02	0.97 (0.92-1.03)	.36	0.99 (0.93-1.04)	.62
**OLS score tertile**						
**1**	Ref		Ref		Ref	
**2**	0.91 (0.83-1.00)	.06	0.98 (0.89-1.08)	.67	0.98 (0.89-1.08)	.66
**3**	0.89 (0.80-1.00)	.05	0.96 (0.86-1.08)	.52	0.97 (0.86-1.09)	.59
**Fine-Gray model[a]**						
**OLS score, per s**	0.95 (0.89-1.01)	.11	0.99 (0.93-1.06)	.73	0.99 (0.94-1.07)	.9
**OLS score tertile**						
**1**	Ref		Ref		Ref	
**2**	0.93 (0.83-1.03)	.9	1.00 (0.90-1.15)	.9	1.00 (0.89-1.11)	.9
**3**	0.91 (0.83-1.03)	.9	0.98 (0.86-1.12)	.9	0.99 (0.87-1.12)	.9

Note: OLS score and high-density lipoprotein cholesterol were log-transformed due to skewed distribution.
[a]In Fine-Gray model, mortality was considered as a competing risk.
Model 1: adjusted for sex and estimated glomerular filtration rate.
Model 2: adjusted for model 2 plus body mass index, systolic blood pressure, chronic obstructive pulmonary disease history, dementia history, diabetes mellitus history, and cardiovascular disease history.
Model 3: adjusted for model 3 plus smoking habit, alcohol consumption, and high-density lipoprotein cholesterol.
Abbreviations: OLS, one-leg stand; HR, hazard ratio; CI, confidence interval; Ref, reference.

Supplementary Table 5. Cause-specific hazard ratios for incident chronic kidney disease according to physical performance test after excluding subjects with comorbidities[a].

	Model 1		Model 2		Model 3	
	**HR (95% CI)**	***P***	**HR (95% CI)**	***P***	**HR (95% CI)**	***P***
TUG score						
Time, per s	1.48 (1.26-1.73)	<.001	1.46 (1.24-1.71)	<.001	1.43 (1.21-1.68)	<.001
OLS score						
Time, per s	0.92 (0.86-0.99)	.03	0.95 (0.88-1.03)	.21	0.97 (0.90-1.04)	.40

Note: TUG and OLS test score, and high-density lipoprotein cholesterol were log-transformed due to skewed distribution.
[a]Comorbidities included chronic obstructive pulmonary disease, dementia, and cardiovascular disease.
Model 1: adjusted for sex and estimated glomerular filtration rate.
Model 2: adjusted for Model 2 plus body mass index, systolic blood pressure, and diabetes mellitus history.
Model 3: adjusted for Model 3 plus smoking habit, alcohol consumption, and high-density lipoprotein cholesterol.
Abbreviations: TUG; timed up and go, OLS; one-leg stand; HR, hazard ratio; CI, confidence interval

Supplementary Table 6. Hazard ratios for incident chronic kidney disease according to timed up and go test after excluding subjects who have reached outcome within 2 years of the physical performance test.

	Model 1		Model 2		Model 3	
	HR (95% CI)	*P*	HR (95% CI)	*P*	HR (95% CI)	*P*
**Cause-specific model**						
**TUG score, per s**	1.30 (1.13-1.50)	<.001	1.25 (1.08-1.44)	.002	1.21 (1.05-1.40)	.01
**TUG score tertile**						
**1**	Ref		Ref		Ref	
**2**	1.09 (0.97-1.22)	.13	1.08 (0.97-1.21)	.16	1.09 (0.97-1.22)	.16
**3**	1.25 (1.10-1.42)	.001	1.19 (1.05-1.36)	.008	1.17 (1.03-1.34)	.02
**Fine-Gray model[a]**						
**TUG score, per s**	1.28 (1.10-1.49)	.002	1.23 (1.05-1.43)	.009	1.21 (1.03-1.41)	.02
**TUG score tertile**						
**1**	Ref		Ref		Ref	
**2**	1.11 (0.98-1.25)	.10	1.10 (0.97-1.24)	.12	1.11 (0.99-1.25)	.08
**3**	1.23 (1.07-1.41)	.004	1.17 (1.02-1.35)	.03	1.16 (1.01-1.34)	.04

Note: TUG score and high-density lipoprotein cholesterol were log-transformed due to skewed distribution.
[a]In Fine-Gray model, mortality was considered as a competing risk.
Model 1: adjusted for sex and estimated glomerular filtration rate.
Model 2: adjusted for model 2 plus body mass index, systolic blood pressure, chronic obstructive pulmonary disease history, dementia history, diabetes mellitus history, and cardiovascular disease history.
Model 3: adjusted for model 3 plus smoking habit, alcohol consumption, and high-density lipoprotein cholesterol.
Abbreviations: TUG, 3-m timed up and go; HR, hazard ratio; CI, confidence interval, Ref, reference.

Supplementary Table 7. Hazard ratios for incident chronic kidney disease according to one-leg stand test after excluding subjects who have reached outcome within 2 years of the physical performance test.

	Model 1		Model 2		Model 3	
	HR (95% CI)	*P*	HR (95% CI)	*P*	HR (95% CI)	*P*
**Cause-specific model**						
**OLS score, per s**	0.92 (0.86-0.98)	.02	0.96 (0.90-1.03)	.24	0.97 (0.90-1.03)	.31
**OLS score tertile**						
**1**	Ref		Ref		Ref	
**2**	0.90 (0.81-1.01)	.08	0.96 (0.86-1.07)	.45	0.96 (0.86-1.08)	.51
**3**	0.86 (0.75-0.99)	.03	0.92 (0.80-1.05)	.24	0.93 (0.81-1.07)	.30
**Fine-Gray model[a]**						
**OLS score, per s**	0.94 (0.87-1.01)	.08	0.98 (0.90-1.05)	.55	0.98 (0.91-1.06)	.9
**OLS score tertile**						
**1**	Ref		Ref		Ref	
**2**	0.92 (0.82-1.04)	.20	0.98 (0.87-1.10)	.72	0.99 (0.87-1.11)	.8
**3**	0.87 (0.75-1.01)	.07	0.93 (0.80-1.08)	.35	0.94 (0.81-1.09)	.44

Note: OLS score and high-density lipoprotein cholesterol were log-transformed due to skewed distribution.
[a]In Fine-Gray model, mortality was considered as a competing risk.
Model 1: adjusted for sex and estimated glomerular filtration rate.
Model 2: adjusted for model 2 plus body mass index, systolic blood pressure, chronic obstructive pulmonary disease history, dementia history, diabetes mellitus history, and cardiovascular disease history.
Model 3: adjusted for model 3 plus smoking habit, alcohol consumption, and high-density lipoprotein cholesterol.
Abbreviations: OLS, one-leg stand; HR, hazard ratio; CI, confidence interval, Ref, reference.

Supplementary Table 8. ICD-10 codes used for comorbidity detection.

Comorbidities	Definitions	ICD-10 Codes or Procedure codes
Myocardial infarction	Defined from diagnosis*	ICD-10: I21, I22, I25.2
Hypertension	Composite of following: >140/90 mmHg at baseline examination one or more ICD codes (I10-13 or I14) with antihypertensive two or more ICD codes prior to baseline examination	ICD-10: I10, I11, I12, I13, I15 plus all kinds of oral antihypertensive
Diabetes mellitus	Defined from diagnosis* plus treatment	ICD-10: E10, E11, E12, E13, E14 Treatment: all kinds of oral antidiabetics and insulin
Peripheral arterial disease	Defined from diagnosis*	ICD-10: I70, I71
Congestive heart failure	Defined from diagnosis*	ICD-10: I11.0, I50, I97.1
Malignancy	Defined from diagnosis*	ICD-10: C00-C97
Chronic obstructive pulmonary disease	Defined from diagnosis* plus treatment	ICD-10: J42, J43(except J43.0), J44 Treatment: SABA, SAMA, LABA, LAMA, ICS, ICS+LABA, or methylxanthine (>1 months).
Dementia	Defined from diagnosis*	ICD-10: F00, F03, G30, G31
History of renal replacement therapy	Defined from hemodialysis or peritoneal dialysis, claim code or operation of kidney transplantation	Korean procedure codes: O7010, O7020, O7061, O7062, O9991

Note: * To ensure accuracy, comorbidities were established based on one inpatient or two outpatient records of ICD-10 codes in the database

Abbreviations: ICD-10, 10th revision of the International Statistical Classification of Diseases and Related Health Problems; SABA, Short acting beta agonists; SAMA, short acting muscarinic antagonist; LAMA, long acting muscarinic antagonist; LABA, long acting beta agonists; ICS, Inhaled corticosteroids.
